# Highly Specific and Sensitive Detection of *Yersinia pestis* by Portable Cas12a-UPTLFA Platform

**DOI:** 10.3389/fmicb.2021.700016

**Published:** 2021-07-07

**Authors:** Yang You, Pingping Zhang, Gengshan Wu, Yafang Tan, Yong Zhao, Shiyang Cao, Yajun Song, Ruifu Yang, Zongmin Du

**Affiliations:** State Key Laboratory of Pathogen and Biosecurity, Beijing Institute of Microbiology and Epidemiology, Beijing, China

**Keywords:** lateral flow immunochromatographic assay, up-converting phosphor technology, nucleic acid detection, *Yersinia pestis*, Cas12a

## Abstract

The recent discovery of collateral cleavage activity of class-II clustered regularly interspaced short palindromic repeats–CRISPR-associated protein (CRISPR-Cas) makes CRISPR-based diagnosis a potential high-accuracy nucleic acid detection method. Colloidal gold-based lateral flow immunochromatographic assay (LFA), which has been combined with CRISPR/Cas-based nucleic detection, usually associates with drawbacks of relative high background and the subjectivity in naked-eye read-out of the results. Here, we developed a novel system composed of Cas12a-based nucleic acid detection and up-converting phosphor technology (UPT)-based LFA (UPT–LFA), termed Cas12a-UPTLFA. We further demonstrated the utility of this platform in highly sensitive and specific detection of *Yersinia pestis*, the causative agent of the deadly plague. Due to high infectivity and mortality, as well as the potential to be misused as bioterrorism agent, a culture-free, ultrasensitive, specific, and rapid detection method for *Y. pestis* has long been desired. By incorporating isothermal recombinase polymerase amplification, the Cas12a-UPTLFA we established can successfully detect genomic DNA of *Y. pestis* as low as 3 attomolar (aM) and exhibited high sensitivity (93.75%) and specificity (90.63%) for detection of spiked blood samples with a detection limit of 10^2^ colony-forming unit per 100 μl of mouse blood. With a portable biosensor, Cas12a-UPTLFA assay can be operated easily by non-professional personnel. Taken together, we have developed a novel Cas12a-UPTLFA platform for rapid detection of *Y. pestis* with high sensitivity and specificity, which is portable, not expensive, and easy to operate as a point-of-care method. This detection system can easily be extended to detect other pathogens and holds great promise for on-site detection of emerging infectious pathogens.

## Introduction

Recently, several promising clustered regularly interspaced short palindromic repeats (CRISPR)-based diagnostic systems called Specific High Sensitivity Enzymatic Reporter UnLOCKing (SHERLOCK), one-HOur Low-cost Multipurpose highly Efficient System (HOLMES), DNA endonuclease-targeted CRISPR trans reporter (DETECTR), and Cas14-DETECTR (Gootenberg et al., 2017; Chen et al., 2018; Harrington et al., 2018; Li et al., 2018) were established. These diagnostic systems are based on the collateral nuclease activity of CRISPR-associated proteins (Cas), which allows for the sequence-specific detection of nucleic acids via reporter nucleic acid degradation, as initially demonstrated for Cas13 (East-Seletsky et al., 2016). For instance, the class II type V-A Cas12a effector was reported to cleave double-stranded DNA (dsDNA) by utilizing RuvC catalytic domain under the guidance of the sequence-specific crRNA. Cas12a recognizes a unique protospacer-adjacent motif (PAM) sequence of TTTN, and the formation of a ternary complex of Cas12/crRNA/target DNA is required for the collateral cleavage activity. Activated Cas12a nucleases indiscriminately cleave the single-stranded DNA (ssDNA) reporter that is labeled with the fluorophore and fluorophore quencher at the both sides, leading to emitting a detectable fluorescent signal (Chen et al., 2018). These methods have the potential to fit all the affordable, sensitive, specific, user-friendly, rapid and robust, equipment-free, and deliverable to end-users (ASSURED) criteria for nucleic acid detection proposed by the World Health Organization (Peeling et al., 2006). To date, CRISPR/Cas-based detections coupled with colloidal gold strip have been developed to be point-of-care testing (POCT) tools and employed to detect many pathogens, such as Ebola, Lassa, Zika, dengue, human papillomavirus (HPV), and severe acute respiratory syndrome coronavirus 2 (SARS-CoV-2) (Chen et al., 2018; Gootenberg et al., 2018; Barnes et al., 2020; Patchsung et al., 2020).

**FIGURE 1:**
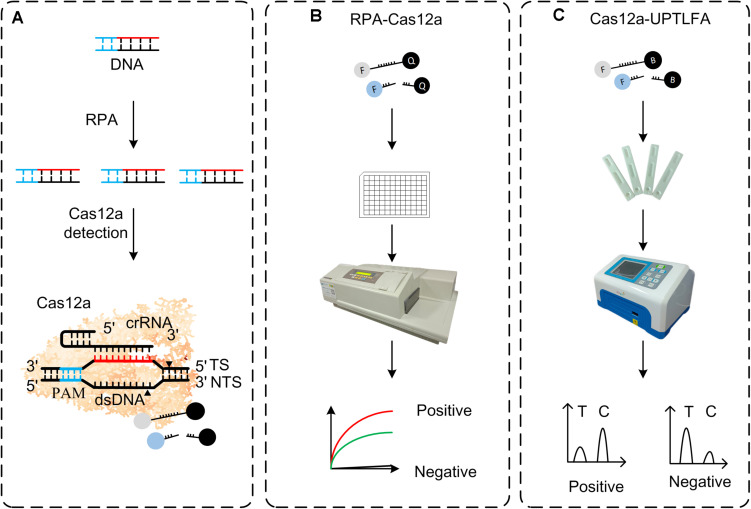
Overview of RPA-Cas12a and Cas12a-UPTFLA platform. **(A)** Target genes were amplified by RPA prior to Cas12a detection. Upon recognition of their dsDNA targets, Cas12a nucleases were activated, and the reporters were then cleaved indiscriminately. **(B)** FAM-BHQ reporters were used in RPA-Cas12a detection by fluorescence read-out method using a microplate reader. A positive sample will exhibit rapid increase of fluorescence signals, while a negative sample will only produce background signal. **(C)** UPT lateral flow immunochromatographic assay was incorporated with RPA-Cas12a detection method described in panel **(B)** to be Cas12a-UPTFLA. This method adopted FAM-biotin, instead of FAM-BHQ, labeled ssDNA as the reporter, and results were obtained by scanning using an UPT biosensor. Cutoff value was set according to the T/C values of the negative controls to designate the positive or negative results. RPA, recombinase polymerase amplification; UPT, up-converting phosphor technology.

However, colloidal gold-based lateral flow assay (LFA) usually associates with the drawbacks of relative high background and false-positive rates, as well as the subjectivity in naked-eye read-out of the results (Gootenberg et al., 2018; Patchsung et al., 2020). Up-converting phosphor technology (UPT) is based on lanthanide-containing Up-converting phosphor nanoparticle (UCP) that can absorb infrared light and emit visible light. Biological matrices usually do not have this unique up-convert feature (Corstjens et al., 2005), which makes UCPs optical reporter superior than gold nanoparticles. Detection method using UCP-labeled conjugates has much less background noise than the complicated biological samples, further increasing its the sensitivity and decreasing the false-positive rate of detection. UPT-LFA employs UCP as bioconjugates and has been developed for detection of many substrates, including bacterial pathogens (Corstjens et al., 2008; Zhang et al., 2014, 2020; Liang et al., 2017), toxins (Zhao et al., 2016), drugs (Hu et al., 2018), and clinical biomarkers (Yang et al., 2017). In order to improve the performance of CRISPR-based LFA method, we use UCP as bioconjugates to develop a portable detection platform for nucleic acid (Figure 1).

Rapid and sensitive detection of *Yersinia pestis*, a Tire-1 biothreat agent that causes the plague known as the “Black Death,” is of critical importance for plague surveillance and prevention. Sporadic plague outbreaks are reported every year; and outbreaks of plague in Madagascar, India, Democratic Republic of Congo, China, and Mongolia warn us that plague is not an eradicated disease (Gushulak et al., 1994; Bazanova et al., 2009; Li et al., 2013; Abedi et al., 2018; Andrianaivoarimanana et al., 2019). Culturing bacteria from samples to improve the detection rate is impossible for in-field detection, especially for the highly virulent pathogens including *Y. pestis* due to the biosafety concern. Therefore, this study aimed to establish a culture-free, highly specific, and sensitive CRISPR/Cas-based UPT-LFA (Cas12a-UPTLFA) platform for *Y. pestis* detection. After incorporation of isothermal recombinase polymerase amplification (RPA), this platform could successfully detect *Y. pestis* genomic DNA at as low as 3 attomolar (aM) and 1 colony-forming unit per microliter (CFU/μl) *Y. pestis*-spiked blood samples. This is at least 10-fold sensitive than the conventional F1 antigen-based UPT-LFA detection of *Y. pestis*, which has a detection limit of 10^4^ CFU/ml for pure culture of *Y. pestis* EV76 (Yan et al., 2006). With a portable UPT biosensor, Cas12a-UPTLFA can be performed by non-professional personnel in 1.3 h as a POCT method, without the need of expensive quantitative PCR instruments or microplate reader. In preparation of our manuscript, Schultzhaus et al. (2021) reported a Cas13a-based nucleic acid detection method that enables detection of *Y. pestis lcrV* gene with attomole sensitivity, which is comparable with our results. Our study represents the first application of CRISPR/Cas12a-based nucleic acid detection combined with UPT-LFA, which holds promise to be a rapid, sensitive, specific diagnostic platform easily extended to detection of emerging infectious diseases in the future.

## Materials and Methods

### Purification of Cas12a Protein

pMBP-LbCas12a was a gift from Jennifer Doudna (Addgene plasmid # 113431) (Chen et al., 2018). The vector was transformed into BL21 (DE3) competent cells. Bacterial cells containing Cas12a expression vector were induced by 0.5 mM of isopropyl β-D-1-thiogalactopyranoside (IPTG) at 16°C for 12–14 h until OD_600 nm_ approximately reached 0.6. Bacterial cells were harvested by centrifugation at 9,820*g* for 15 min and resuspended in lysis buffer containing 50 mM of NaH_2_PO_4_, 300 mM of NaCl, and 10 mM of imidazole (pH 7.5). After ultrasonication, the supernatant of the bacterial lysate was separated, and Cas12a protein was purified by Ni-NTA Agarose (Qiagen, Hilden, Germany). After 1.5 h TEV (New England Biolabs, Ipswich, MA, United States) cleavage at 30°C, the his-MBP-tag proteins were removed by Ni-NTA agarose, and the purified proteins were analyzed by sodium dodecyl sulfate–polyacrylamide gel electrophoresis (SDS-PAGE) (Sangon Biotech, Shanghai, China). The concentration of the purified Cas12a protein was calculated using the following formula: protein concentration (mg/ml) = 1.45 × OD_280 nm_ – 0.74 × OD_260 nm_. The Cas12a protein was diluted to 1 μM in phosphate-buffered saline (PBS), divided into 50 μl of aliquots, and frozen at −80°C for long-term storage.

### Design and Preparation of Primers, crRNAs, and Reporters

Four genes (*pla*, *ymt*, *ypo2088*, and *lcrV*) were chosen for developing the Cas12a-based nucleic detection assay for *Y. pestis*. In order to choose the targeting sites that can induce nuclease activity of Cas12a with high efficiency, the target gene was scanned for regions containing the 5′-TTTN-3 PAM of Cas12a. The full length of crRNA guide sequence was 41 nt including 21-nt repeats and 20-nt target sequence adjacent to a PAM. For each target gene, 3–5 crRNAs were designed and prepared for testing their efficacy. The crRNAs that exhibited the highest efficiency were used in the following detection assay.

The RPA primers were designed according to the manufacturer’s instructions (TwistDx, Cambridge, United Kingdom), and all the primers have a length between 30 and 35 nt with an expected amplicon size of about 300 bp. Reporters labeled with 5(6)-carboxy fluorescein (FAM) and biotin were used in Cas12a-UPTLFA detection, while those labeled with FAM and black hole quencher (BHQ) were used in RPA-Cas12a.

All RPA primers, PCR primers, real-time quantitative PCR (qPCR) primers, and reporters were synthesized commercially (Sangon Biotech, China). Sequences of DNA and RNA are listed in detail in Supplementary Tables 1, 2.

### RPA-Cas12a Detection System

Recombinase polymerase amplification was performed using TwistAmp Basic kit (TwistDx, United Kingdom). Briefly, 1 μl of forward and 1 μl of reverse primers (10 μM), 29.5 μl of rehydration buffer, 2.5 μl of MgOAc (280 mM), 10 μl of DNA template, and 6 μl of RNase-free water were mixed into a 50 μl reaction mixture unless otherwise indicated. The reaction was incubated at 39°C for 18 min.

Cas12a reaction mixture containing 5 μl of CutSmart Buffer (New England Biolabs, United States), 5 μl of crRNA (2 μM), 5 μl of Cas12a (1 μM), 5 μl of reporters (10 μM), 10 μl of RPA product, and 20 μl of RNase-free water were mixed into a 50 μl reaction mixture. The reaction was performed at 37°C for the indicated time course in SpectraMax (Molecular Devices, San Jose, CA, United States) with fluorescence value measured at 1-min interval (λex = 490 nm; λem = 520 nm).

### Optimization of Cas12a Reaction Mixture

*pla* gene was amplified with primers *pla*-F and *pla*-R using the isolated *Y. pestis* genomic DNA as the template and cloned into pUC19 vector, generating plasmid pUC19-pla. For all the optimization of Cas12a reaction, 3 μl of pUC19-*pla* (100 ng/μl) was used as sample. Five microliters of different reaction buffer was used in buffer optimization experiments. For reporter optimization, 5 μl of reporters (10 μM) was added into the reaction mixtures. The detail components of buffer are listed in Supplementary Table 3. For crRNAs screening tests, 3 μl of purified PCR products of four targets was used as samples, and other conditions were same as described in Section “RPA-Cas12a Detection System.”

### Template DNA Preparation

#### For Sensitivity Assay

Genomic DNA was extracted from the overnight culture of *Y. pestis* strain 201 using QIAamp DNA Mini kit (Qiagen, Germany) according to the manufacturer’s instruction. The purified DNA samples were quantified using a NanoDrop 2000 (Thermo Fisher, Waltham, MA, United States) and then diluted in RNase-free water to 3 × 10^7^ aM followed by 10-fold serial dilutions. For sensitivity assay of Cas12a-based fluorescent detection, 3 μl of *Y. pestis* DNA templates of various concentrations was added into the Cas12a reaction mixture mentioned above. For sensitivity assay of RPA-Cas12a detection, 10 μl of *Y. pestis* genomic DNA of various concentrations was added to the RPA reaction mixture for amplification, and then 10 μl of RPA product was transferred into Cas12a reaction mixture.

#### For Specificity Assay

The cultures of *Y. pestis* strain 201 (pMT1^+^, pCD1^+^, and pPCP1^+^), *Yersinia pseudotuberculosis* strain Pa3606 (pYV^+^), and *Yersinia enterocolitica* ATCC9610 (pYV^–^) were prepared to be 10^8^ CFU/ml in PBS (Gibco, Grand Island, NY, United States). Aliquots of 1 ml of different bacterial suspensions were centrifuged at 2,400*g* for 10 min to collect bacterial cells. The supernatant was discarded, and the bacterial cells were then resuspended in 200 μl of ddH_2_O. The resuspended solutions were boiled in a water bath for 10 min, and the supernatants were collected by centrifugation at 13,800*g* for 10 min. Five microliters of the supernatant was added to the reaction mixtures for RPA or PCR amplification.

#### Preparation of *Y. pestis*-Spiked Samples

To prepare the *Y. pestis*-spiked blood and lung samples of mice, 1 ml of PBS suspensions of *Y. pestis* (10^8^ to 10 CFU/ml) was centrifuged at 2400*g* × 10 min, the supernatants were removed, and 100 μl of mouse blood (BioRab, Beijing, China) or 100 μl of mouse lung homogenate (20 mg lung tissue) was added and thoroughly mixed, resulting in *Y. pestis*-spiked blood and lung samples containing the bacteria ranging from 10^8^ to 10 CFU. DNA was extracted using DNeasy Blood and Tissue Kit (Qiagen, Germany) for lung samples and TGuide S96 Magnetic Viral DNA/RNA Kit (TIANGEN Biotech, Beijing, China) for blood samples, and 10 μl of extracted DNA solution was used for RPA reaction. Two microliters of DNA solution was added to the reaction mixtures for qPCR detection.

To prepare the *Y. pestis*-spiked soil samples, 1 ml of PBS suspensions of *Y. pestis* (10^8^ to 10 CFU/ml) was centrifuged at 2,400*g* × 10 min, the supernatants were removed, and then 100 mg of soil was added and thoroughly mixed. DNA was extracted by TIANamp Soil DNA Kit (TIANGEN Biotech, Beijing, China), and 10 μl of extracted DNA solution was used for RPA reaction.

### Fabrication of the UPT-Lateral Flow Strips for RPA-Cas12a Detection

Up-converting phosphor nanoparticle (UCP, NaYF4:Yb3^+^ and Er3^+^) nanoparticles had an average diameter of about 50 nm (Shanghai Kerune Phosphor Technology Co. Ltd., Shanghai, China). The excitation and emission wavelengths of the particles are 980 nm and 541.5 nm, respectively (Zhao et al., 2016; Liang et al., 2017). UCPs were covalently conjugated with the monoclonal antibody against 5(6)-FAM (Biocare, Shanghai, China), and the immunochromatographic lateral flow strips were prepared as previously described (Sun et al., 2016). In brief, the UCP-anti-FAM-mAb conjugate solution was added to the glass fiber (Millipore, Bedford, MA, United States) as a conjugation pad, and then the pad was incubated at −80°C overnight and freeze-dried. Nitrocellulose (NC) membrane (Millipore, Bedford, MA, United States) was coated with a test line of streptavidin (2 mg/ml in 0.01 M of PB; Abcam, Cambridge, MA, United States) and a control line of goat anti-mouse IgG (2 mg/ml in 0.01 M of PB, prepared by our lab) with the microarrayer Iso Flow Dispense (Imagene Technology, Lebanon, NH, United States) at a rate of 1 μl/cm. The coated NC membrane was dried at 37°C for 1 h. Finally, the sample pad, conjugate pad, NC membrane, and absorbent paper (Shanghai Goldbio Technology Co. Ltd., Shanghai, China) were put together on the lamination card (Shanghai Liangxin Biotechnology Company, Shanghai, China) with proper overlaps. Each strip was cut to a width of 4 mm with the Cutting Machine ZQ4000 (Shanghai Goldbio Technology Co. Ltd., China).

For UPT-LFA, RPA-Cas12a reactions were performed as described in Section “RPA-Cas12a Detection System,” except that 5 μl of FAM-biotin-labeled ssDNA (5 μM) was used, instead of FAM-BHQ-labeled ssDNA. Three microliters of product solution of RPA-Cas12a assay was added into 240 μl of sample-treating buffer and mixed thoroughly. Then, 100 μl of mixtures was applied to the UPT strips, which were scanned by a UPT-3A biosensor (Beijing Hotgen Biotech Co., Beijing, China) after 15 min.

### qPCR and PCR

Real-time quantitative PCR was performed in a total volume of 20 μl of mixture containing 10 μl of 2 × Premix Ex Taq^TM^ (TaKaRa, Dalian, China), 0.4 μl of forward and 0.4 μl of reverse primers (10 μM), 0.8 μl of Taqman probe (10 μM), 2 μl of DNA template, and 6.4 μl of ddH_2_O. Real-time PCRs were performed in a LightCycler 480 instrument (Roche, Basel, Switzerland). The reaction was subjected to an initial denaturation at 95°C for 30 s, followed by 40 cycles of denaturation at 95°C for 5 s, annealing and extension at 60°C for 30 s, and a final extension at 50°C for 30 s.

Polymerase chain reaction was performed in a total volume of 50 μl of mixture containing 25 μl of 2 × Taq PCR MasterMix (Biomed, Wuhan, China), 1 μl of forward and reverse primers (10 μM), 5 μl of DNA template, and 19 μl of ddH_2_O. PCRs were performed by T100^TM^ Thermal Cycler (BIO-RAD, Hercules, CA, United States) using the following program: initial denaturation at 95°C for 5 min, then 95°C for 30 s, 58°C 40 s, and 72°C 10 s for 35 cycles. For *ypo2088*, the annealing temperature was 62°C.

## Results

### Optimization of Cas12a-Based Nucleic Acid Detection of *Y. pestis*

Generally, most of *Y. pestis* strains possess a single circular chromosome and three plasmids termed pCD1, pPCP1, and pMT1. Detection methods relying solely on one plasmid or chromosomal gene may yield a false-negative result or a non-specific signal due to the close genetic relatedness of *Y. pestis* to the other species in the genera *Yersinia*, especially for the human pathogenic *Yersinia pseudotuberculosis* and *Yersinia enterocolitica*, which cause widely distributed but self-limited zoonotic diseases. In this study, we include three plasmid-encoded genes (*pla* in pPCP1, *ymt* in pMT1, and *lcrV* in pCD1) and a chromosomal gene *ypo2088* that codes for a *Y. pestis*-specific putative methyltransferase (Bai et al., 2020), to ensure the accuracy and the specificity of the detection. Next, Cas12a reaction mixtures were prepared as described in Section “RPA-Cas12a Detection System” using the purified protein (Supplementary Figure 1) and to test the efficacy of the detection system. Consistent with the previous studies, the detection sensitivity of Cas12a detection system was low for all the four target genes, when no pre-amplification was performed. Among the four target genes, the most sensitive performance was obtained for *pla* detection. When using *Y. pestis* genome DNA (3 × 10^4^ fM) as the samples to be detected, about 300 relative fluorescence unit (RFU) signal intensity could be yielded, and no positive signal was found for the samples at lower concentrations (Supplementary Figure 2). No distinct signal has been detected for *ymt* and *ypo2088* at all the tested concentrations, whereas only very faint signals have been detected for *lcrV* gene. These results are reasonable since about 150 to 200 copies of the pPCP1 encoding *pla* gene are present per *Y. pestis* bacterium (Parkhill et al., 2001).

To enhance the sensitivity, we optimized the Cas12a-mediated DNA detection system. Previous studies indicated that Cas effectors possess biases to the different homopolymeric reporters and that different buffer compositions would influence their nuclease activity (Gootenberg et al., 2018; Teng et al., 2019). We next tested the cleavage preference of Cas12a-crRNA complex on various homopolymer reporters. The results showed that Cas12a preferred to cut ploy-C reporter, followed by poly-A and poly-T reporters sequentially (Supplementary Figure 3) when using *pla* as the target gene. Therefore, poly-C reporter was used in the following experiments. Then, we evaluated the influence of different buffer on the efficiency of the detection system. Based on the cleavage efficiency and availability (Supplementary Figure 4), CutSmart Buffer (NEB) was chosen to be reaction buffer. Finally, we evaluated the efficiency of various targets or crRNAs (Supplementary Figure 5); synthesized crRNA with the highest efficiency for each target gene was adopted for our Cas12 detection system.

**FIGURE 2:**
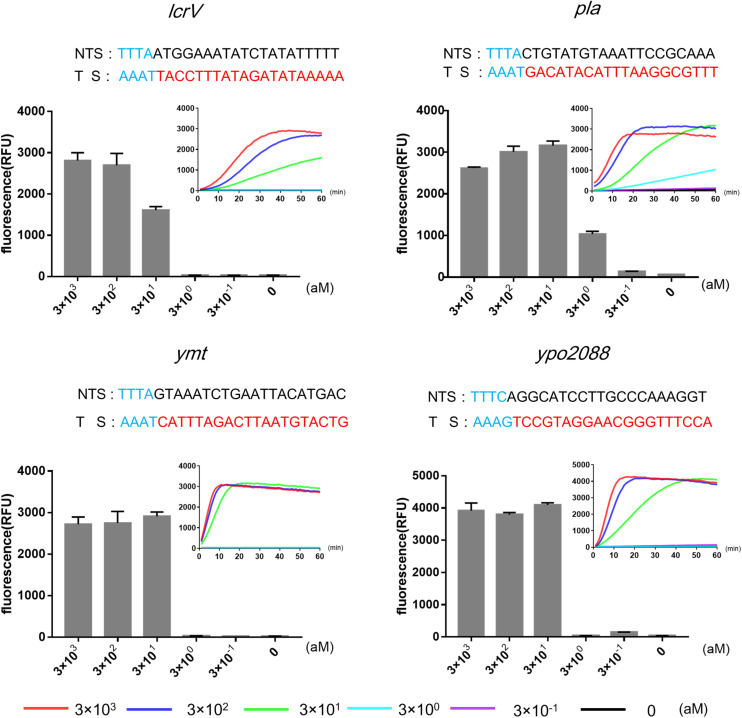
Limits of detections (LODs) of RPA-Cas12a detection of four target genes of *Yersinia pestis* using different concentrations of genomic DNA (3 × 10^− 1^ to 3 × 10^3^ aM). Real-time curves show fluorescence signals taken every minute within 1 h. Bar graphs show the endpoint fluorescence intensities detected at 1 h of detection. Data are represented as mean with standard deviation (SD) (*n* = 3). LODs, limits of detection; RPA, recombinase polymerase amplification.

Next, we used RPA, an isothermal amplification method, to amplify the target genes prior to Cas12a detection. We determined the limit of detection (LOD) of RPA-Cas12a assay using *pla* as the target gene, and *Y. pestis* genomic DNA as low as 3 aM (corresponding to 1.8 copies/μl) could be reliably detected, resulting in more than 10^7^-fold higher sensitivity than the detection by Cas12a alone, similar to that of qPCR (Supplementary Figure 6). For the detection of *ymt*, *lcrV*, or *ypo2088* genes, the LODs were about 30 aM, 10-fold lower than those of the detection of *pla* (Figure 2).

### Specificity of RPA-Cas12a Detection

**FIGURE 3:**
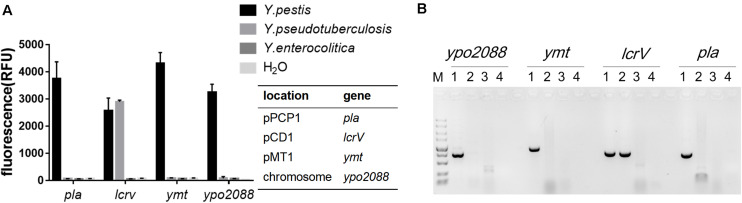
The specificity of the RPA-Cas12a detection of *Y. pestis*. **(A)** Specificity of RPA-Cas12a. Data are represented as mean with SD (*n* = 3). **(B)** Specificity of PCR assay for the same DNA templates used in panel **(A)**. M indicates DNA molecular marker; 1–4 represent *Y. pestis* strain 201, *Yersinia pseudotuberculosis* strain Pa3606, *Yersinia enterocolitica* ATCC9610, and the negative control, respectively. RPA, recombinase polymerase amplification.

*Yersinia pestis*, *Y. pseudotuberculosis*, and *Y. enterocolitica* are the closely related pathogenic species within the genus *Yersinia*, and they are easily confused with each other in nucleic acid detection due to the genetic similarities. In general, *Y. pestis*, *Y. pseudotuberculosis*, and *Y. enterocolitica* share a common 70-kb plasmid (termed as pCD1 in *Y. pestis* and pYV in the other two species), while pMT1 and pPCP1 are unique to *Y. pestis*. Here, we used *Y. pseudotuberculosis* Pa3606 (pYV^+^) and *Y. enterocolitica* ATCC9610 (pYV^–^) to evaluate the specificity of the RPA-Cas12a assay for *Y. pestis*. DNA extracted from the three bacterial strains was RPA-amplified and subjected to detection. Fluorescence signals were detected only in *Y. pestis* templates but not in the other two strains when using *pla*, *ymt*, or *ypo2088* as the target. In addition, *lcrV* was detected in both the *Y. pestis* and *Y. pseudotuberculosis* templates, because they both possess a pCD1/pYV1 plasmid, which were also consistent with the PCR results (Figure 3). These results demonstrated that RPA-Cas12a detection exhibited high specificity and can easily discriminate the closely related *Yersinia* bacteria. Actually, a specific and positive result for any of the chromosome gene *ypo2088*, or genes encoded by plasmids unique to *Y. pestis*, i.e., *pla* in pPCP1 and *ymt* in pMT1 plasmids, suggests that the tested samples contain *Y. pestis* bacteria or its DNA. A combination of the detection results for all of the four target genes can provide detailed information on the *Y. pestis* strain contained in samples, such as which virulence plasmids are possessed by the strain. In the following experiment, we used *pla* gene as the target gene to further evaluate the performance of our method.

### Detection of Environmental and Biological Samples Spiked With *Y. pestis* in by RPA-Cas12a

**FIGURE 4:**
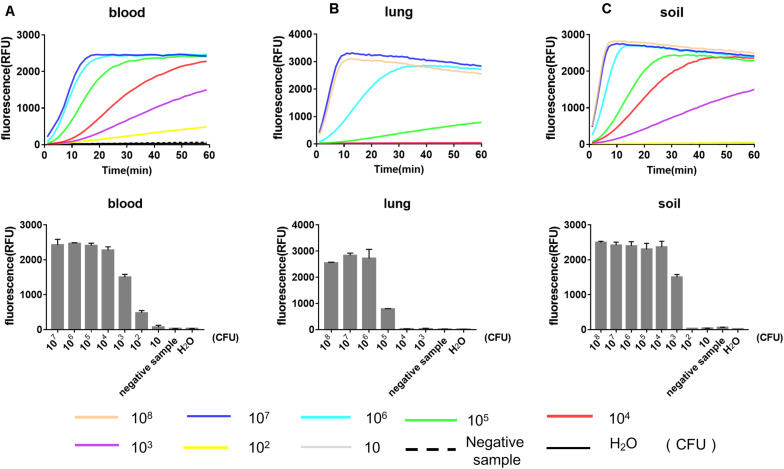
Performance of RPA-Cas12a for the detection of *Y. pestis*-spiked environmental and tissue samples. Results for the mouse blood samples **(A)**, lung tissues **(B)**, and soil samples **(C)** spiked with *Y. pestis* ranging from 10^1^ to 10^7^ CFU. For preparation of the spiked samples, 100 μl of mouse blood, 100 μl of lung homogenate (containing 20 mg of mouse lung tissues), and 100 mg of soil were used as the sample matrix. Real-time curve shows the fluorescence signals taken every minute within 1 h. Bar graphs show the endpoint fluorescence intensities detected at 1 h of detection. Data are represented as mean with SD (*n* = 3). RPA, recombinase polymerase amplification.

We further evaluated the performance of our RPA-Cas12a system for the detection of *Y. pestis*-spiked environmental and mouse tissues samples (Figure 4). Soil samples were artificially contaminated with a serial numbers of *Y. pestis* bacterium, and DNA was extracted and analyzed as described above. The LOD of our RPA-Cas12a system for the spiked soil samples was 10^3^ CFU of *Y. pestis* per 100 mg of soil, suggesting good tolerance of our detection system to the complicated matrix in soil. To evaluate the efficacy of RPA-Cas12a system for the spiked biological samples, different numbers of *Y. pestis* bacteria cells were added and mixed thoroughly with 100 μl of lung homogenate (20 mg of lung tissue of mice) or 100 μl of mouse blood. As low as 10^2^ CFU of *Y. pestis* per 100 μl of blood could be reliably detected, whereas at least 10^5^ CFU of *Y. pestis* per 20 mg of tissues could be detected by the same method, suggesting that the interference of lung tissue for the RPA-Cas12a assay was relatively high (Figure 4). The high sensitivity of RPA-Cas12a system for *Y. pestis*-spiked blood sample is meaningful, since clinical blood samples are easily accessed and the bubonic plague often progresses to secondary septicemia, which is often associated with a high number of bacteria in blood.

### Incorporation of UPT-LFA Into RPA-Cas12a Detection of *Y. pestis*

**FIGURE 5:**
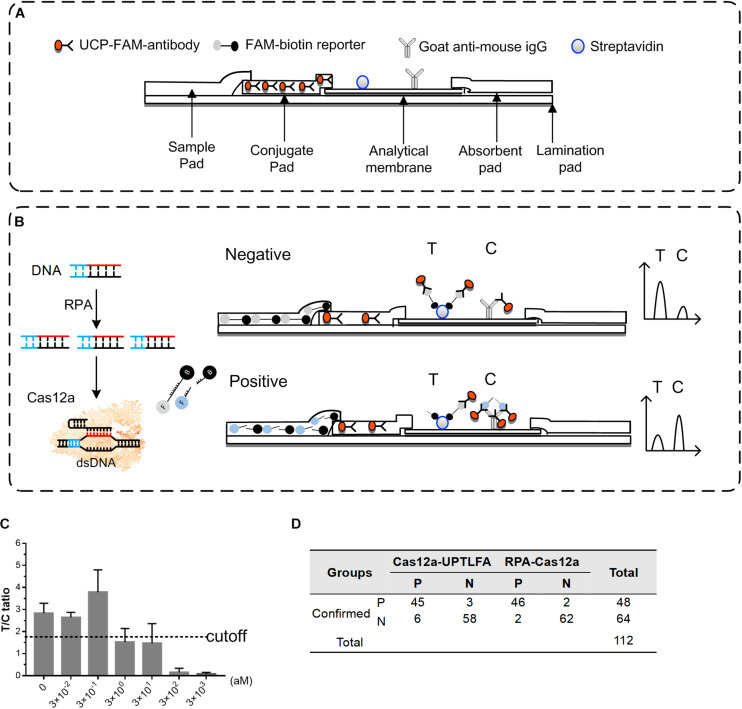
Performance of Cas12a-UPTLFA on detection of the *Y. pestis* genomic DNA or *Y. pestis* bacteria in the spiked blood samples. **(A)** Components of the Cas12a-UPTLFA platform. The sample pad, conjugation pad, analytical membrane, and absorbent pad were mounted on a lamination card with proper overlaps. All antibodies for detection were fixed in advance, including the UCP-labeled with anti-FAM antibody in the conjugation pad, streptavidin for the T line, and goat anti-mouse IgG for the C line. **(B)** Cas12a-UPTLFA detection relies on the cleavage of a FAM-biotin-labeled ssDNA reporter by the collateral activity of the Cas12 enzymes upon target recognition, allowing the detection on UPT strips. **(C)** Detection results of *Y. pestis* genomic DNA samples at concentration ranging from 3 × 10^− 2^ to 3 × 10^3^ aM. Data are represented as mean with SD (*n* = 3). **(D)** The table shows the summarized results for 112 blood samples detected by Cas12a-UPTLFA platform and RPA-Cas12a fluorescent read-out assay. P, the number of positive samples; *N*, the number of negative samples; RPA, recombinase polymerase amplification; UPT, up-converting phosphor technology.

Using the FAM-biotin-labeled ssDNA as the reporter, instead of FAM-BHQ-labeled ssDNA used above, we managed to incorporate UPT-LFA into the RPA-Cas12a system, termed Cas12a-UPTLFA. The strip was fabricated as previously described (Zhao et al., 2016). UCPs covalently coated with anti-FAM antibody via the aldehyde group were present in the conjugation pad (Figure 5A). Streptavidin and goat anti-mouse IgG were arranged on NC membrane as test (T) and control (C) line, respectively (Figure 5B). The signal derived from UCPs at T and C lines were defined as T and C values for each of the tested samples, whereas the T/C ratio represents the detection results. Concretely, when the RPA-Cas12a reaction product of the negative sample was applied to the strip, abundant FAM-biotin-labeled reporters will firstly bond with the UCP-labeled anti-FAM antibody in the conjugation pad and then accumulate at the T line via binding to streptavidin, reducing the amount of UCP-labeled anti-FAM bond to the goat anti-mouse IgG on the C line, and the T/C ratio maintains a high level. For the positive samples, cleavage of reporters will reduce the accumulation of UCP-labeled anti-FAM antibody at the T line and results in increasing signals on the C line, and the T/C ratio will decrease significantly (Figure 5B).

For the optimization of Cas12a-UPTLFA platform, when RPA-Cas12a product containing 0.5 μM of FAM-biotin reporter was mixed with the sample-treating buffer at 1:80 ratio, significant and stable signals can be generated for the known positive samples. Therefore, this procedure was adopted in the following experiments. The performance of Cas12a-UPTLFA for the detection of *Y. pestis* genomic DNA samples was further evaluated. The samples without the addition of the genomic DNA were used as negative controls. The average of T/C ratio of the negative controls minus 2SD was set as the cutoff value for the designation of positive or negative results (Figure 5C). When Cas12a-UPTLFA platform was applied for the detection of *Y. pestis* genomic DNA, the lowest concentration that could be detected was 3 aM, similar to that of the fluorescent read-out based RPA-Cas12a assay, as well as the qPCR results using primers specific for *pla* gene (Figure 2 and Supplementary Figure 6).

To evaluate the performance of Cas12a-UPTLFA on the *Y. pestis*-spiked samples, a batch of commercial mouse blood ware equally divided into 112 portions, and 64 of them were left untreated and 48 were spiked with *Y. pestis* bacterium ranging from 10^2^ to 10^7^ CFU (eight replicates for each concentration), which served as the negative and positive samples, respectively (Figure 5D). All the 112 blood samples were subjected to DNA extraction and detection by Cas12a-UPTLFA or RPA-Cas12a fluorescent read-out assay. The sensitivity of Cas12a-UPTLFA and RPA-Cas12a fluorescent read-out assay was 93.75% (45/48) and 95.83% (46/48), and the specificity was 90.63% (58/64) and 96.88% (62/64), respectively. The detection limits of the two methods were both 10^2^ CFU per sample, and the 75% (6/8) positive samples could be successfully detected at the lowest detection concentration for both of the methods. The same batch of spiked blood samples was subjected to qPCR analysis of *pla* gene, and we found that the lowest concentration that can be detected was 100 CFU per sample, the same with the detection limits of Cas12a-UPTLFA (Supplementary Figure 7).

## Discussion

Although different methods have been reported for the nucleic acid detection of both virus and bacterial pathogens including *Y. pestis*, various limitations still exist for the rapid, deployable, and field detection of these highly contiguous agents because of the requirement of expensive instruments or professional personnel to perform complex operation. Here, we provide an alternative method for the POCT detection of pathogens. By combining UPT-LFA with RPA-Cas12a detection, a high sensitivity and specificity diagnostic platform, termed Cas12a-UPTLFA, was firstly established. Due to the generality of our UPT lateral flow strips, they can be prefabricated in batch and the Cas12a-UPTLFA can easily be adapted to detect any emerging infectious pathogens by introducing different crRNA and RPA primers specific for the interested target nucleic acids. The advantage of this platform is obvious when comparing with the antigen-based rapid diagnostic test, for which there is often a lag time to create a sensitive antibody for the detection, while pathogen-specific crRNA and RPA primers can be easily designed and obtained by commercial synthesis with low prices. For in-field application of Cas12a-UPTLFA, the operation simplicity for users is obvious; and procedures of RPA pre-amplification, Cas12a reaction, and UPT-LFA require only minimal technical proficiency. With a portable UPT biosensor, detection of *Y. pestis* with a sensitivity similar to that of the laboratory testing can be achieved in about 1.3 h by Cas12a-UPTLFA, including 18 min for RPA, 45 min for Cas reaction, and 15 min for UPT assay.

The performance of our Cas12a-UPTLFA was evaluated for the detection of the cultured bacterial samples, as well as *Y. pestis*-spiked environmental and biological samples. The detection limit of Cas12a-UPTLFA was 3 aM for the isolated genomic DNA using *Y. pestis*-specific gene *pla* as the target, equal to 1.8 genome copy per microliter (18 copies per test), which is comparable with the 2 aM of synthetic Zika and Dengue ssRNA targets by SHERLOCKv2 (Gootenberg et al., 2018) and slightly higher than the 42 RNA copies per reaction for SARS-CoV-2 in clinical samples detected by a Cas13-based SHERLOCK (Patchsung et al., 2020). For the detection of *Y. pestis*, this result is more sensitive than a published study using isothermal DNA amplification methods (RPA, thermophilic helicase-dependent isothermal DNA amplification, and LAMP) combined with LFA to detect the biothreat agents including *Y. pestis*, in which the detection limit was 100–1,000 genome copies for all the target bacteria (Zasada et al., 2018). It was also more sensitive than the conventional F1 antigen-based UPT-LFA detection of *Y. pestis*, which has a detection limit of 10^4^ CFU/ml (10^3^ CFU per test) for pure culture of *Y. pestis* EV76 (Yan et al., 2006). The gold standard for nucleic acid detection, qPCR, has been recently reported to achieve a higher sensitivity of 1 fg, equal to 0.18 copy genomes per test, for the detection of *Y. pestis pst* gene by using pentaplex real-time PCR (Bai et al., 2020). When Cas12a-UPTLFA is applied to the complicated biological samples, i.e., blood sample spiked with *Y. pestis*, as low as 1 CFU/μl concentration can be reliably detected. In the detection of 112 spiked blood samples, Cas12a-UPTLFA showed the same detection limit with that of RPA-Cas12a fluorescent read-out assay, as well as similar sensitivity and specificity, indicating that our system has the potential to be a portable, culture-free, highly specific, and sensitive method for plague epidemiological surveillance in the epidemic areas.

## Data Availability Statement

The original contributions presented in the study are included in the article/Supplementary Material, further inquiries can be directed to the corresponding authors.

## Author Contributions

ZD and RY conceived the project, supervised the project, and designed the experiments. YY, PZ, GW, YT, YZ, SC, and YS performed the experiments. ZD, RY, YY, and PZ analyzed the data and wrote the manuscript. All authors contributed to the article and approved the submitted version.

## Conflict of Interest

The authors declare that the research was conducted in the absence of any commercial or financial relationships that could be construed as a potential conflict of interest.
