# Supplementary material for: Highly Specific and Sensitive Detection of Yersinia pestis by Portable Cas12a-UPTLFA Platform
Source: Front Microbiol. 2021 Jul 7;12:700016. doi: 10.3389/fmicb.2021.700016 (PMC8292961; doi:10.3389/fmicb.2021.700016)
Supplement: Supplementary file 1 [file Data_Sheet_1.docx]

***Supplementary information***

**Supplementary Figures**

**
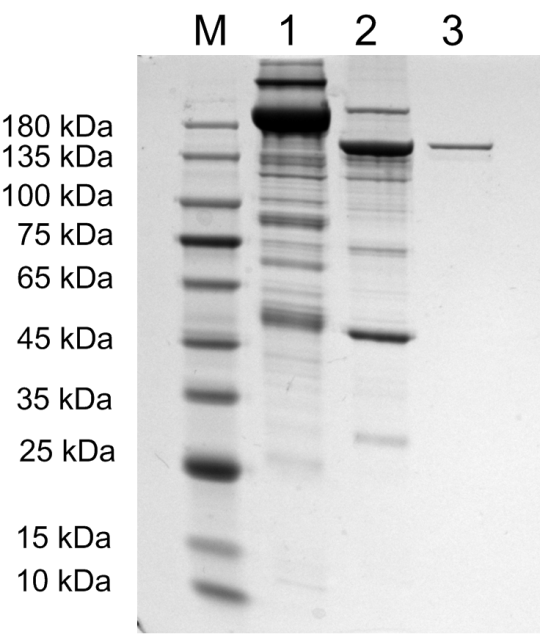
**

**Supplementary Figure 1.** SDS-PAGE analysis ofpurified Cas12a. M indicates protein molecular marker; lane 1 represents His-MBP-taggedCas12apurified using Ni-NTA Agarose; lane 2 representsrecombinantCas12a protein after treatment with TEV protease; lane 3represents the purifiedCas12a proteinafter removing of the His-MBP tag.Specifically, 40 μL of protein samples were mixed with 10 μL 5×SDS loading buffer, and incubated for 10 mins in a boiling water bath. 10 μLof samples were analyzed by 15% SDS-PAGE (Sangon Biotech, China) in running buffer (50 mM HEPES, 0.1% SDS, 2 mM EDTA) at 160 volts.


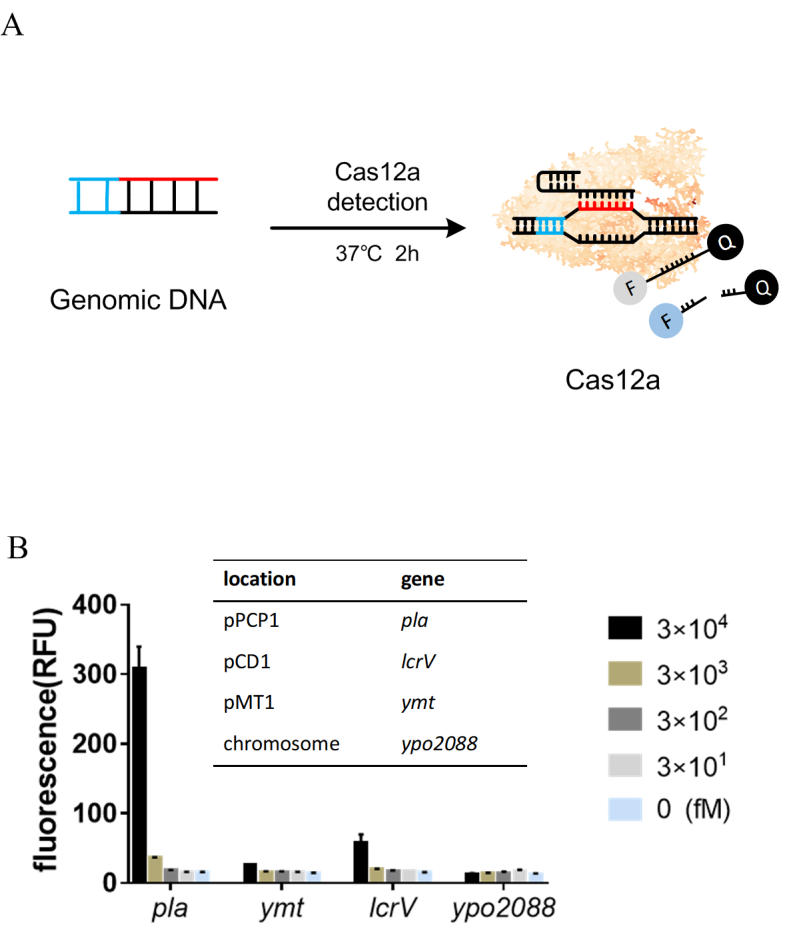


**Supplementary Figure 2**. Performance of Cas12a alone detection. (A) Schematic view of Cas12a detection of genomic DNA. (B) LOD of Cas12a detection of four target genes of *Y. pestis*was determined using genomic DNA at different concentrations (3×10^1^to 3×10^4^fM)*.* Bar graphs showedthe endpoint fluorescence intensities after the 2 hours incubation. Data are represented as mean with standard deviation(SD)(n=3).


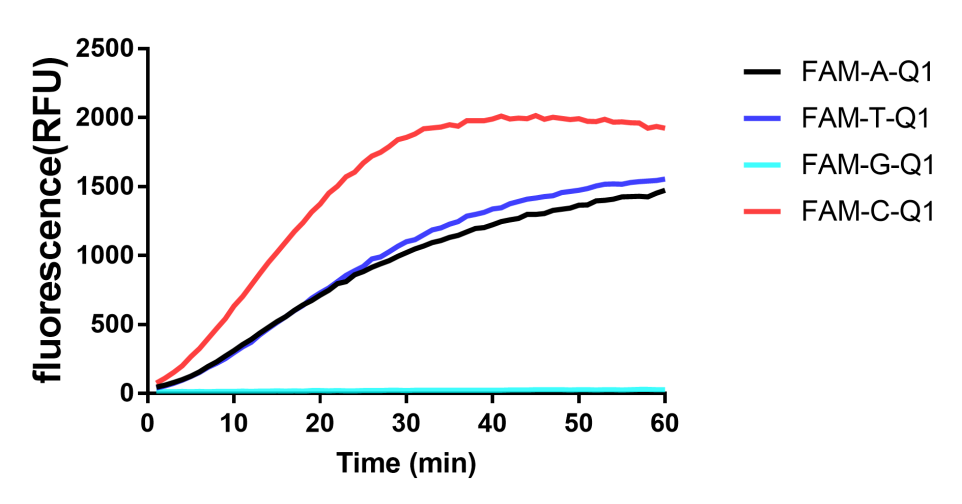


**Supplementary Figure 3.** Optimization of the reporter used in fluorescence-read out Cas12a detection. ssDNA reporters containing a homoploymer of A, T, G or C bases were analyzed.


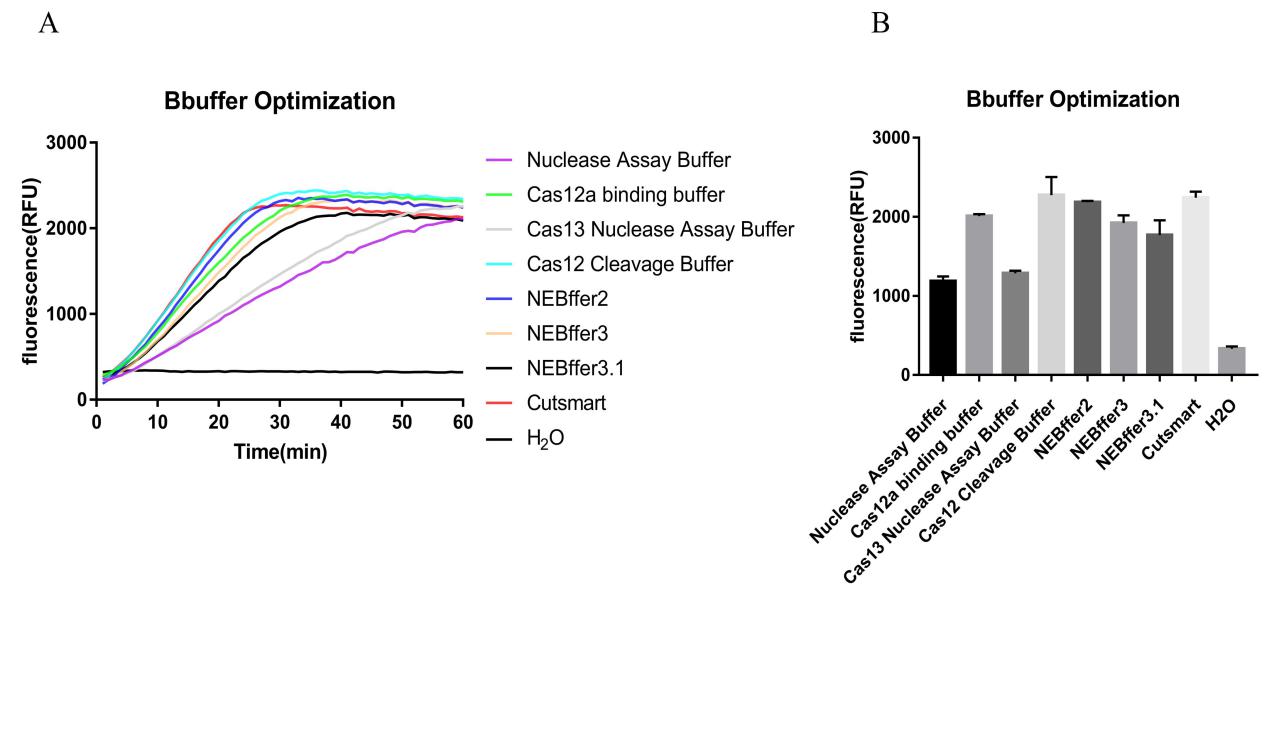


**Supplementary Figure 4**. Optimization of reaction buffer for Cas12a detection. Buffers of different compositionswere tested for their performance on Cas12a alone detection of *pla*gene using pUC19-plaassamples. (A) Real-time curvesof fluorescence signals using different reaction buffer. (B) Bar graphs showedthe endpoint fluorescence intensitiesdetected at30 min for the different reaction buffer. Data are represented as mean with SD(n=3).


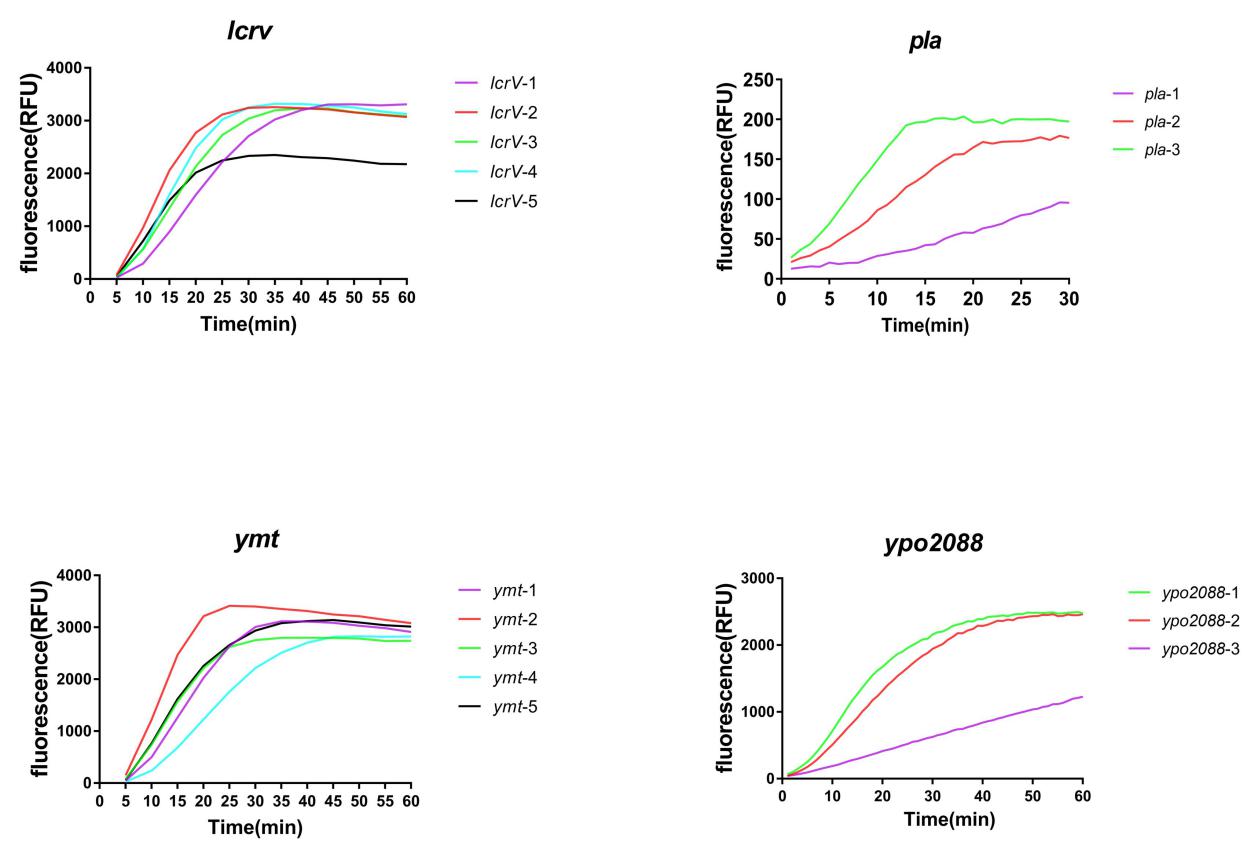


**Supplementary Figure 5.** Screening the optimized crRNA for the four target genes used in this study.3μL of PCR products were added into the Cas12a reaction mixtures. The reactionswere performed at 37 °C andreal-time curves showed the fluorescence signals taken every minute within 1 hourusingSpectraMax.


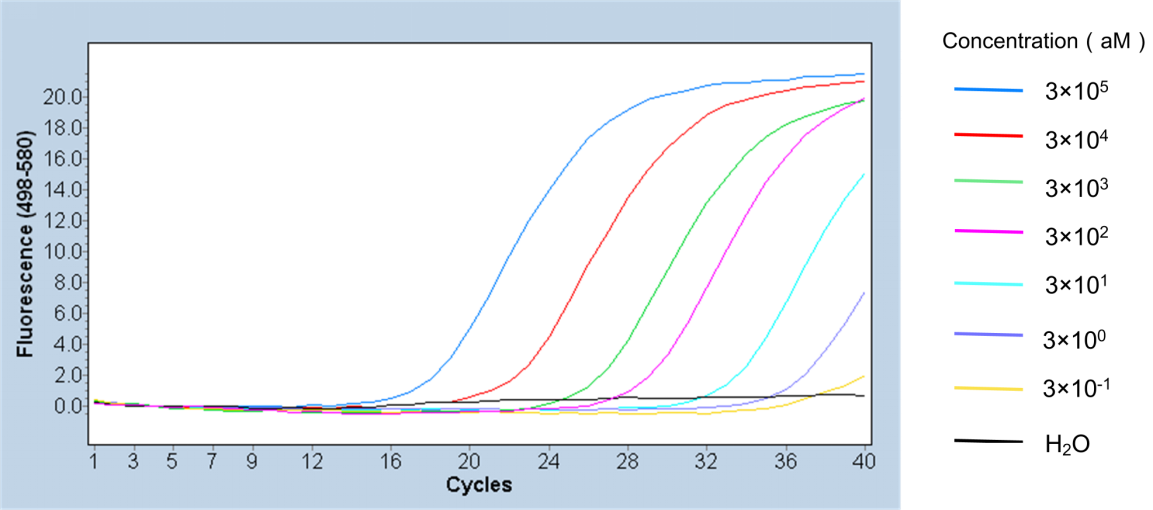


**Supplementary Figure 6.** LOD of qPCR for the detection of *pla*in*Y. pestis* genomic DNA samples. The concentrations of *Y. pestis* genomic DNA were from 3×10^-1^ to 3×10^5^aM.


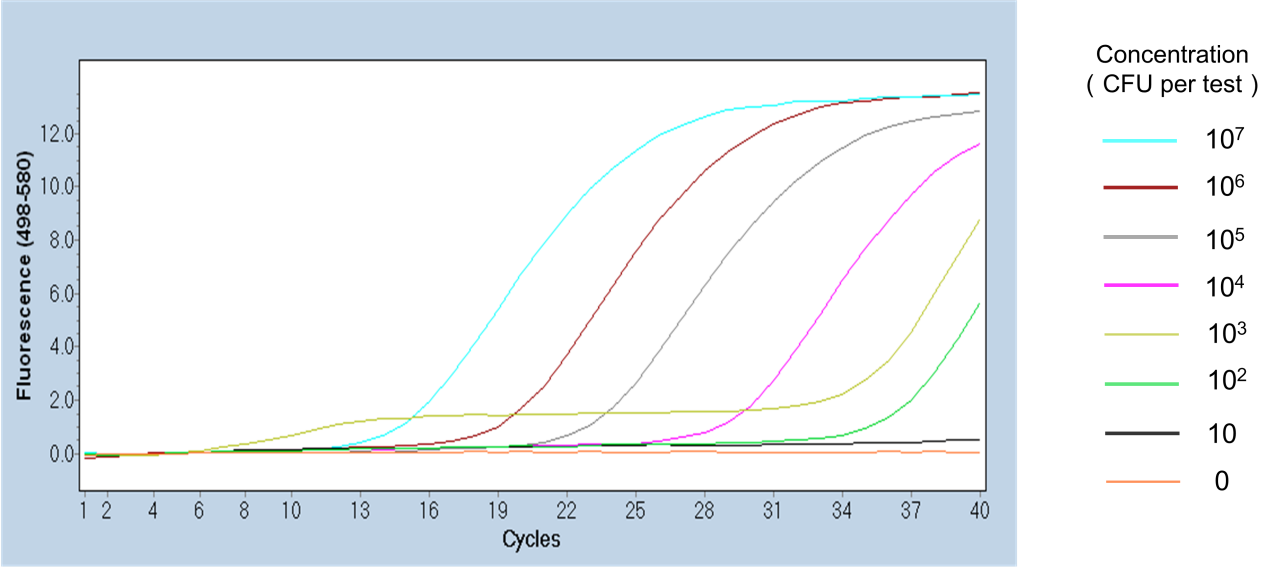


**Supplementary Figure7.** LOD of qPCR for the detection of *pla*in*Y. pestis*spiked blood samples.The concentrations of *Y. pestis* were from 10^7^ to 10 CFU per 100 μL blood.

**SupplementaryTables**

**Supplementary Table1．Nucleotide sequence of primers and reporters used in this study.**

| Name | Sequence  （5’ to 3’） | Usage |
| --- | --- | --- |
| pla-f | GATTACGCCAAGCTTGAGATTAAGGGTGTCT | Construction of pUC19-*pla* |
| pla-r | GTACCCGGGGATCCGGGAGGATGAAAAGAG | Construction of pUC19-*pla* |
| pla-Taq-f | GTAATAGGTTATAACCAGCGCTT | Real time PCR assay of *pla* |
| pla-Taq-r | AGACTTTGGCATTAGGTGTG | Real time PCR assay of *pla* |
| pla-Taq-probe | FAM-ATGCCATATATTGGACTTGCAGGCCAGT-BHQ1 | Real time PCR assay of *pla* |
| pla-PCR-f | GTCCGGGAGTGCTAATGCAG | PCR amplification of *pla* |
| pla-PCR-r | GTCTGAGTACCTCCTTTGCCC | PCR amplification of *pla* |
| ymt-PCR-f | ACGTGTATCCTGATTTCCCACC | PCR amplification of *ymt* |
| ymt-PCR-r | CTTGATGCGGGGGTTTTTCC | PCR amplification of *ymt* |
| lcrV-PCR-f | AGCCTACGAACAAAACCCACA | PCR amplification of *lcrV* |
| lcrV--PCR-r | GGACTTATCCGAGCAGGTGG | PCR amplification of *lcrV* |
| yop2088-PCR-f | ATGACACTGCGTGAATGCCT | PCR amplification of *ypo2088* |
| ypo2088-PCR-r | TACTGCGTTGTTGGCCTCAG | PCR amplification of *ypo2088* |
| ypo2088-f | AAATTAGTCATCAGGGCAACAATGGGATTTGGTTC | RPA amplification of *ypo2088* |
| ypo2088-r | CGTTGAAACTGTGTCATGGTCTAACATGCATTGG | RPA amplification of *ypo2088* |
| pla-f | ATATAGTTATAATAATGGAGCTTATACCGGAAACT | RPA amplification of *pla* |
| pla-r | TATTCTTATCAATGGTCTGAGTACCTCCTTTG | RPA amplification of *pla* |
| lcrV-f | TGCGAGGGCAAATTATTTAATATGATTAGAGCCTA | RPA amplification of  *lcrV* |
| lcrV-r | ACGCCCGCAATTCCCATTGTGTATTCGGCGATGAT | RPA amplification of *lcrV* |
| ymt-f | TGTATCCTGATTTCCCACCAATCAACGATACAAGA | RPA amplification of  *ymt* |
| ymt-r | TTTCATGCAAGTTGAGTAGGTCCTCTTGCCGTTGC | RPA amplification of *ymt* |
| FAM-C**-**BHQ1 | FAM-CCCCC-BHQ1 | Cas12a detection reporter  Cas12a detection reporter |
| FAM-T**-**BHQ1 | FAM-TTTTT-BHQ1 |  |
| FAM-A**-**BHQ1 | FAM-AAAAA-BHQ1 | Cas12a detection reporter |
| FAM-G**-**BHQ1 | FAM-GGGGG-BHQ1 | Cas12a detection reporter |

**Supplementary Table2**．**Nucleotide sequence of crRNA used in this study**

| Name | Sequence （5’ to 3’） |
| --- | --- |
| ymt-crRNA-1 | UAAUUUCUACUAAGUGUAGAUAAAGCAACAAAGTCAGGGGA |
| ymt-crRNA-2* | UAAUUUCUACUAAGUGUAGAUGTAAATCTGAATTACATGAC |
| ymt-crRNA-3 | UAAUUUCUACUAAGUGUAGAUAGCGGATCTTCAGGCTTATC |
| ymt-crRNA-4 | UAAUUUCUACUAAGUGUAGAUTACTCTTCCATGTTTTCGTA |
| ymt-crRNA-5 | UAAUUUCUACUAAGUGUAGAUCCTACTGATAAAACTCTGTC |
| lcrV-crRNA-1 | UAAUUUCUACUAAGUGUAGAUTCTTTGACTAACTGAACCAA |
| lcrV-crRNA-2* | UAAUUUCUACUAAGUGUAGAUATGGAAATATCTATATTTTT |
| lcrV-crRNA-3 | UAAUUUCUACUAAGUGUAGAUAGAATGGCATCCTCGGGTAG |
| lcrV-crRNA-4 | UAAUUUCUACUAAGUGUAGAUCTCGCTTGATGCCATTTTG |
| lcrV-crRNA-5 | UAAUUUCUACUAAGUGUAGAUATTCGGCGGTAAGCTCAGCT |
| ypo2088-crRNA-1* | UAAUUUCUACUAAGUGUAGAUAGGCATCCTTGCCCAAAGGT |
| ypo2088-crRNA-2 | UAAUUUCUACUAAGUGUAGAUCTGCTTTTGCCAGCCTGTAA |
| ypo2088-crRNA-3 | UAAUUUCUACUAAGUGUAGAUCCTAATGTTTTGTCTGGGAG |
| pla-crRNA-1 | UAAUUUCUACUAAGUGUAGAUUAAUUCUCAUCCUGGAGUAA |
| pla-crRNA-2 | UAAUUUCUACUAAGUGUAGAUACUCAAAAUCAUUAAUGCGA |
| pla-crRNA-3* | UAAUUUCUACUAAGUGUAGAUCUGUAUGUAAAUUCCGCAAA |

***：c**rRNAs finally used inRPA-Cas12a and Cas12a-UPTFLA platform were labeled withasterisk.

**Supplemental Table 3. component of different reaction buffer**

| Name | Component |
| --- | --- |
| Nuclease Assay Buffer | 60 mM NaCl, 40 mM Tris-HCl, 6 mM MgCl_2_, pH7.5 |
| Cas12a binding buffer | 20 mM Tris-HCl, 5 mM MgCl_2,_ 100 mM KCl, 1 mM DTT, 5% Glycerol, 50 µg/mL HeparinpH7.5 |
| Cas13 Nuclease Assay Buffer | 60 mM NaCl, 20 mM HEPES, 6 mM MgCl_2_, pH6.8 |
| Cas12 Cleavage Buffer | 150 mM KCl, 20 mM HEPES, 10 mM MgCl_2_, 0.5 mM DTT, 1% Glycerol, pH7.5 |
| NEBffer2 | 50 mM NaCl, 10 mM Tris-HCl, 10 mM MgCl_2_, 1 mM DTT, pH7.9 |
| NEBffer3 | 100 mM NaCl, 50 mM Tris-HCl, 10 mM MgCl_2_, 1 mM dithiothreitol pH 7.9 |
| Cutsmart | 50mM Potassium Acetate, 20mM Tris-acetate, 10mM Magnesium Acetate, 100μg/ml BSA, pH 7.9 |
| NEBffer3.1 | 100 mM NaCl, 50 mM Tris-HCl, 10 mM MgCl_2_, 100 μg/ml BSA, pH 7.9 |
